# Sinus Node Dysfunction and Acute Transverse Myelitis As Initial Presentation of Systemic Lupus Erythematosus in a 55-Year-Old Female: A Case Report

**DOI:** 10.7759/cureus.33957

**Published:** 2023-01-19

**Authors:** Rio May E Llanes

**Affiliations:** 1 Department of Internal Medicine, Chong Hua Hospital, Cebu City, PHL

**Keywords:** case report, methylprednisolone, systemic lupus erythematosus, acute transverse myelitis, sinus node dysfunction

## Abstract

Systemic lupus erythematosus (SLE) is an autoimmune disease involving various organ systems. However, some of these lupus manifestations are underreported but life-threatening, so these unusual presentations need to be documented. This study aims to report a case of sinus node dysfunction (SND) and acute transverse myelitis (ATM) as the initial presentation of SLE.

A 55-year-old Filipina newly diagnosed with SLE initially presented with progressive left upper extremity weakness and numbness within two days. On admission, the patient was noted to have 3/5 left upper extremity weakness and progressive C4-C6 dermatome paresthesia. A computed tomography scan of the brain was negative for infarction or hemorrhage. However, on magnetic resonance imaging of the spine, an ill-defined focus of enhancement was noted from C1 to C4 and extensive edema extending from C1 to mid-C6 vertebra. ATM was considered; hence high dose of intravenous methylprednisolone was given for five days with a notable improvement in motor and sensory deficits. Patients within the same admission also developed an onset of atrial fibrillation in rapid ventricular response in the background of baseline sinus bradycardia with associated episodes of fatigue. SND, attributed to SLE, persisted despite steroids. Pacemaker insertion was done as definitive management. The patient was discharged with the improvement of motor strength to 4+/5 and with prednisone and hydroxychloroquine as discharge medications. In conclusion, recognition and documentation of SLE's rare but life-threatening presentations, such as SLE-ATM and SND, are essential to facilitate timely therapeutic management.

## Introduction

Systemic lupus erythematosus (SLE) is an autoimmune disorder with multiorgan involvement. Cardiac and vascular manifestations have been extensively documented in the available literature such as pericarditis, myocarditis, valvular, coronary, and conduction disorders [1,2]. Conduction system disorders, however, have less known prevalence and yet bear serious implications as these are frequent causes of sudden cardiac death among SLE patients [3-5]. While tachyarrhythmias have been determined in prevalence studies, bradyarrhythmias, particularly sinus node dysfunction (SND), remain less well known [4,6-7]. In case reports described so far among SLE patients with SND, most have associated systemic symptoms such as fever, and myalgia, and its occurrence is likewise associated with a high disease activity [7-9]. Despite the largely undetermined mechanisms behind arrhythmias in SLE, sustained SND is managed with pacemaker insertion [8,10].

On the other hand, acute transverse myelitis (ATM) as a neurologic manifestation is estimated to present in 1%-2% of SLE cases [11]. This can present as a late complication and can manifest as progressive paraparesis, quadriparesis, paresthesia, and incontinence [12,13]. Treatment of SLE-ATM is less well-known owing to the limited cases in current literature. However, most cases would respond to high-dose steroids and immunosuppressants [12,14,15].

In this case report, we describe a 55-year-old female who initially presented with SLE-ATM and SND, and who subsequently improved with immediate initiation of high-dose steroid pulse therapy and pacemaker insertion.

## Case presentation

A 55-year-old previously healthy Filipina presented with weakness of the left upper extremity, initially at the wrist and forearm, which eventually progressed after two days as weakness and numbness now involving the left shoulder. Other than these acute symptoms, the patient would only note intermittent episodes of generalized fatigue for six months. There was no associated dizziness, headache, syncope, facial asymmetry, slurring of speech, alopecia, fever, weight loss, chest pain, palpitations, dyspnea, photosensitivity, rashes, joint pains or bleeding. Blood pressure taken was 180/80 mmHg. The patient initially sought consult at a local hospital and was prescribed clonidine, clopidogrel, and atorvastatin. Persistence of weakness prompted admission to our institution for further work-up. The patient was a known hypertensive for 17 years with fair compliance to irbesartan + amlodipine. She was a non-diabetic, non-smoker, and non-alcoholic beverage drinker. She had her menopause at 48 years old. She had no prior history of extremity weakness and stroke. Her family history was unremarkable for myopathy, autoimmune or neurologic disorders.

On admission, the patient was awake, oriented, and conversant. Blood pressure was 170/90 mmHg, heart rate was 46 beats/min, respiratory rate was 20 cycles/min, and temperature was 36.8 ^o^C. On physical examination, 3/5 motor strength of the distal muscle group of the left upper extremity was noted. Sensation to pain, temperature, and light touch was diminished on the C6 dermatomal distribution, i.e., left thumb and radial aspect of the left index finger. Deep tendon reflexes were normo-reflexive. The rest of the neurologic and systemic physical examinations were unremarkable.

**Figure 1:**
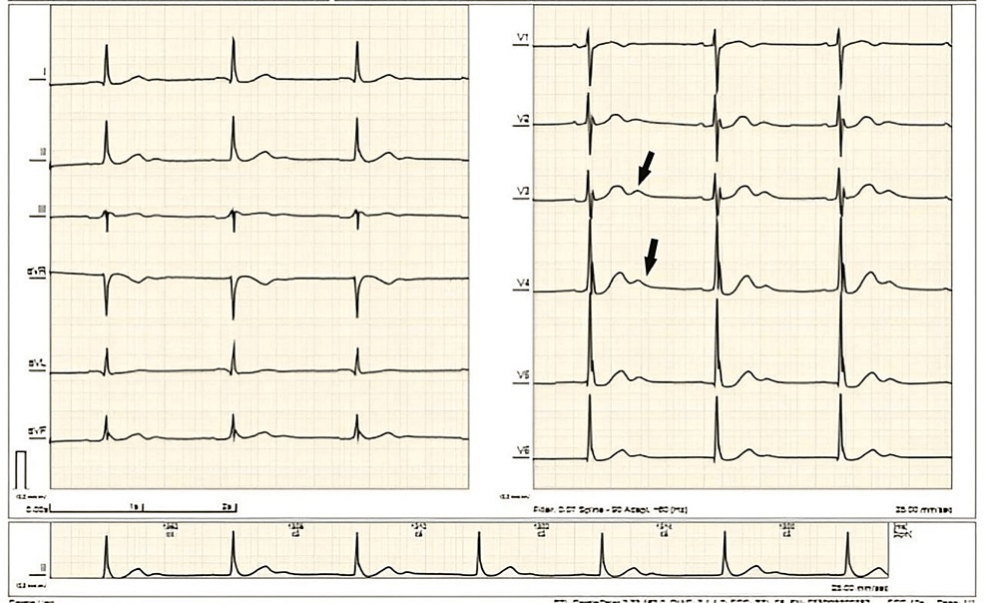
Electrocardiogram of the patient showed sinus bradycardia with intraventricular conduction delay, nonspecific ST-T-wave changes, and prominent U waves (black arrows).

Cerebrovascular disease was initially considered; hence a computed tomography (CT) scan of the brain plain was taken, which showed no evidence of infarction or hemorrhage. A 12-lead ECG (Figure 1) showed sinus bradycardia with intraventricular conduction delay, nonspecific ST segment-T-wave changes, and prominent U waves. Chest radiograph showed clear lung fields and cardiomegaly with no evidence of pulmonary congestion.

Complete blood count (CBC) taken showed normocytic, normochromic anemia with hemoglobin of 9.4 g/dL and hematocrit of 28.4% (Table 1). No leukopenia and thrombocytopenia were noted. The rest of the blood work-up showed normal values except for hypokalemia of 3.1 mmol/L, which was corrected with potassium chloride tablets. Urinalysis was negative for pyuria, hematuria, or proteinuria.

**Table 1:** Pertinent laboratory work-up of reported patient. Abbreviations: CBC - Complete blood count; RBC - Red blood cells; MCV - Mean corpuscular volume; MCH - Mean corpuscular hemoglobin; MCHC - Mean corpuscular hemoglobin concentration; WBC - White blood cells; UIBC - Unsaturated iron binding capacity; TIBC - Total iron binding capacity; BUN - Blood Urea Nitrogen; HBA1C - Glycated hemoglobin; ALT - Alanine aminotransferase; TSH - Thyroid stimulating hormone; FBS - Fasting blood sugar; VLDL - very low-density lipoprotein; LDL - low-density lipoprotein; HDL - high-density lipoprotein; ESR - erythrocyte sedimentation rate; INR - international normalized ratio; PT - prothrombin time; PTT - partial thromboplastin time; H - High; L - Low

Laboratory test	Result	Reference Range
Hemoglobin	9.4 L	12.0 -16.0 g/dL
Hematocrit	28.4 L	37.0 – 47.0%
RBC	3.08 L	4.2 – 5.4 10^6^/uL
MCV	92.2	81 – 99 fL
MCH	30.4	27.0 – 31.0 pg
MCHC	32.9	33.0 – 37.0 g/dL
Platelets	536	130 – 400 10^3^/uL
WBC	5.26	4.8 – 10.8 10^3^/uL
Neutrophil	50.9	40 – 74%
Lymphocyte	39.2	19 – 48%
Monocyte	8.7	3.4 – 9.0%
Eosinophil	0.9	0.0 – 7.0%
Basophil	0.3	0.0 – 1.5%
Serum iron	11.8	8.8-27.0 umol/L
UIBC	39.8	20-62 umol/L
TIBC	51.6	44.8-73.4 umol/L
Ferritin	1006 H	13 – 150 ng/ml
Coomb’s test (Direct)	Negative	Negative
Coomb’s test (Indirect)	Negative	Negative
BUN	16.7	7 – 18 mg/dL
Creatinine	1.0	0.6 – 1.5 mg/dL
Sodium	140	134-148 mmol/L
Potassium	3.1 L	3.3-5.3 mmol/L
HbA1C	4.3	4.8 – 5.9%
ALT	51	5-50 U/L
TSH	1.35	0.30-5.0 uIU/ml
FBS	87	60-100 mg/dL
Cholesterol	155	130-200 mg/dL
Triglycerides	92	60-130 mg/dL
VLDL	18.4	0-50 mg/dL
LDL	104	0-150 mg/dL
HDL	32.6	35-65 mg/dL
ESR	40 H	0-20 mm/hr
PT (Patient)	14.4	
PT Activity	85%	> 70%
PT INR	1.11	< 1.21
PTT (Patient)	28	26.4-36.7 sec.
Bleeding time	5 mins. 53 secs.	2.3 -9.5 mins.

**Figure 2:**
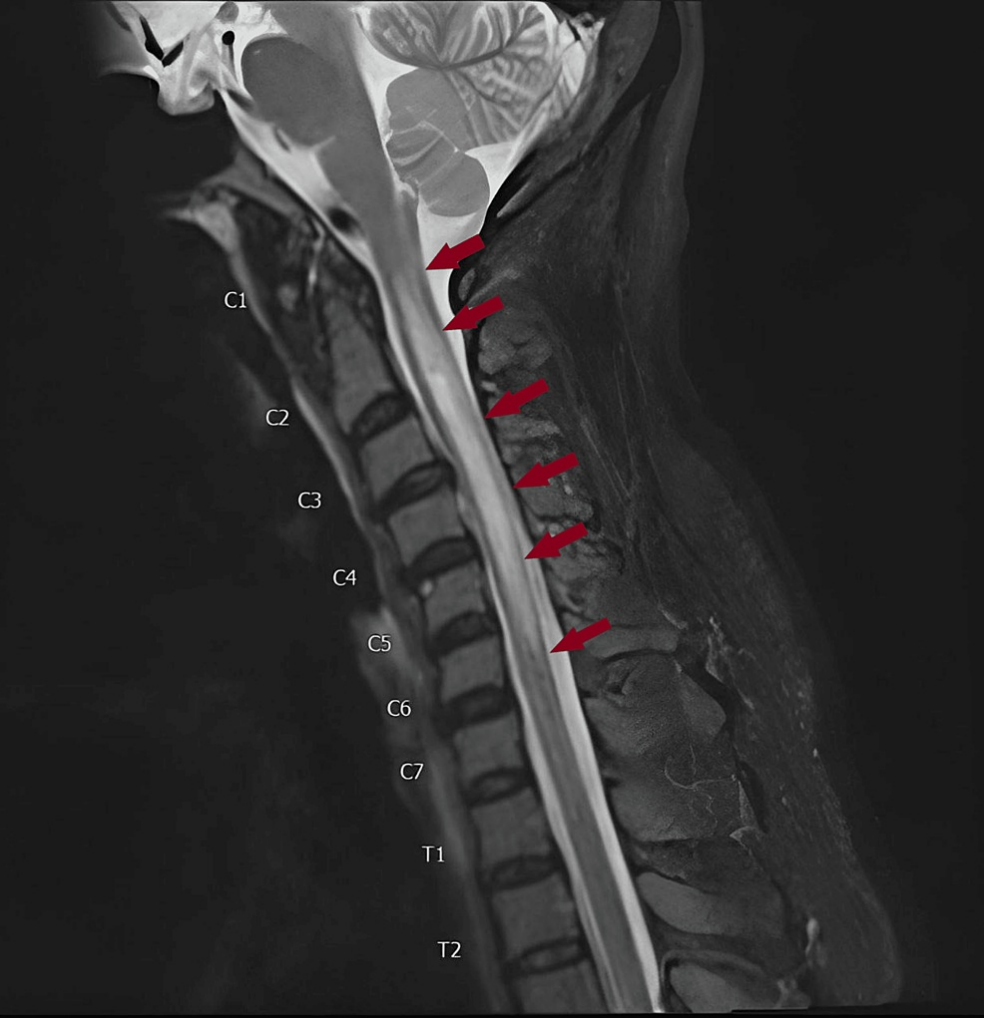
Sagittal T2-weighted image of the cervical spinal cord MRI demonstrating a focus of enhancement from C1 to C4, measuring 8 x 7 x 45 mm with ill-defined margins. Extensive edema extending from C1 to mid-C6 vertebra can also be appreciated (red arrows).

Magnetic resonance imaging (MRI) of the brain was also negative; however, MRI spine screening (Figure 2) showed a bright signal intensity from C1 to the lower endplate of C5, seen on Turbo Inversion Recovery Magnitude (TIRM) and T2-weighted images, comparable with transverse myelitis. MRI of the cervical spine with contrast confirmed the presence of the ill-defined focus of enhancement from C1 to C4, measuring 8 x 7 x 45 mm with ill-defined margins. This was noted to be primarily affecting the left side of the cervical spinal cord with extensive edema extending from C1 to mid C6 vertebra. These findings were compatible with transverse myelitis.

The patient underwent lumbar tap with note of clear, non-turbid, and non-bloody spinal fluid. Opening pressure was low. Cerebrospinal fluid (CSF) studies showed a slightly elevated WBC of 6 mm3 with a lymphocytic predominance (86%). CSF total protein was slightly elevated at 69.5 mg/dL, and glucose was within the normal level. CSF gram stain and culture (bacterial and fungal), acid-fast bacilli, potassium hydroxide, and cryptococcal antigen latex agglutination system (CALAS) tests were negative. CSF meningitis panel and oligoclonal bands were negative.

Further work-up of the patient's anemia included iron studies and bleeding parameters (bleeding time, PT, PTT), which were within normal levels. Coomb's test (direct and indirect) and fecal occult blood test (FOBT) were negative. However, the antinuclear antibody test - immunofluorescence (Table 2) yielded a positive result (up to 1:160 dilution, homogenous pattern). Lupus work-up was pursued, which showed C3 hypocomplementemia and normal C4. Anti-dsDNA showed elevated IgG (39.3 IU/mL) and normal IgM level (0.5 IU/mL). The patient, at this point, met the 2012 Systemic Lupus Collaborating Clinics (SLICC) criteria (neurologic involvement, positive ANA-IF, anti-dsDNA, and low complement) but was one point short of meeting the cut-off for the 2019 European League Against Rheumatism/American College of Rheumatology (ACR/EULAR) classification criteria (nine points) for SLE.

**Table 2:** SLE immunologic work-up of reported patient. Abbreviations: C3 - component 3; C4 - component 4; dsDNA - double-stranded deoxyribonucleic acid; IgG - Immunoglobulin G; IgM - Immunoglobulin M

Laboratory test	Result	Reference Range
Antinuclear antibody- *immunofluorescence (ANA-IF)*	up to 1:160 dilution, homogenous pattern	Negative
Serum Complement C3	0.8 L	0.9-1.0 G/L
Serum Complement C4	0.17	0.1-0.4 G/L
Anti-dsDNA IgG	39.3 H	20 > IU/ml
Anti-dsDNA IgM	0.5	20 > IU/ml

On post-admission day 2, the patient had brief palpitations and was noted to be tachycardic at 110-130s beats/minute, no associated chest pain or dyspnea. Repeat 12-lead ECG showed atrial fibrillation (AF) in rapid ventricular response (RVR). One dose of Digoxin 0.25 mg IVTT was given and started on enoxaparin 6,000 IU subcutaneously once a day. Heart rate improved to 90s beats/minute with no recurrence of palpitations. On post-admission day 3, the patient noted new numbness from the left supraclavicular area up to the proximal two-thirds of the left upper extremity. Sensation to pain and temperature was now noted to be impaired from C4 to C6 dermatomes, i.e., radial aspects of the left arm and forearm and the left supraclavicular area. No associated increased work of breathing or left-sided lag on chest expansion. The patient was also noted to have a single urinary incontinence episode with no associated fecal incontinence, lower extremity weakness, or paresthesia. The patient was immediately started on methylprednisolone 1 g intravenous infusion daily. Carvedilol was initially ordered to control the heart rate of 90-110 beats/minute but was not given as the patient was noted to have a recurrence of bradycardia at 40-50 beats/minute with no associated symptoms. Repeat 12-lead ECG showed AF in slow ventricular response (SVR). SND was considered at this point, attributed to SLE. The patient was started on doxofylline 200 mg half tablet twice a day. A 24-hour Holter monitoring was done, which showed the following rhythms: 1) sinus bradycardia, 2) sinus pause (six-sec duration interrupted by junctional escape rhythm), 3) paroxysmal AF, both rapid and SVR. The patient was referred to electrophysiology service and was apprised for pacemaker insertion. The rest of the cardiac work-up was done. Two-dimensional echocardiography with doppler showed an ejection fraction of 64%, concentric left ventricular hypertrophy with adequate contractility and systolic function, and grade one diastolic dysfunction. Carotid duplex scan and transcranial ultrasound were both unremarkable.

The patient completed five days of high-dose steroid therapy, and the prednisone tablet was started afterward. At this point, there was noted improvement of the left upper extremity motor strength (4+/5), range of motion, and improved numbness of the left shoulder. The patient was subsequently referred to rehabilitation medicine. Heart rate at this time ranged from 40-80 beats/minute, mostly sinus bradycardia but with paroxysmal episodes of AF in SVR.

**Figure 3:**
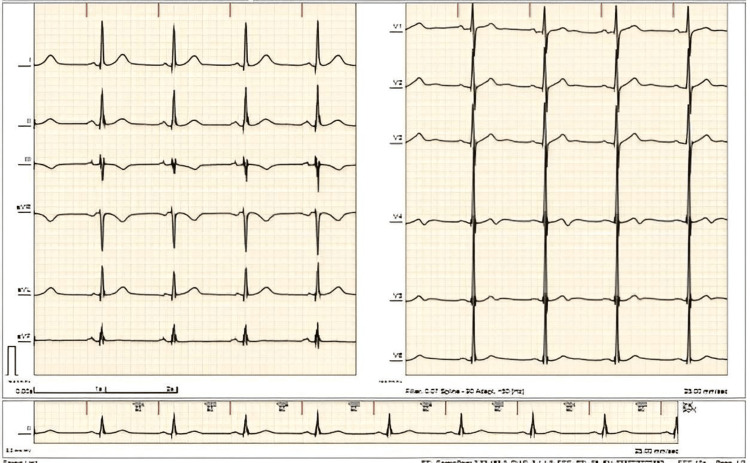
Electrocardiogram of the patient showed atrial paced rhythm with good capture.

The patient eventually consented and underwent dual chamber (DDD) pacemaker insertion on hospital day 14. Subsequent monitoring on telemetry showed an improved heart rate of 60 beats/minute, showing an atrial paced rhythm with good capture (Figure 3). The patient denied fatigue, chest pain, dyspnea or palpitations. She was resumed on prednisone 40 mg one tablet daily and started on hydroxychloroquine 200 mg one tablet daily. The patient remained clinically stable with no symptoms and was eventually discharged.

## Discussion

SLE is a chronic autoimmune disorder that affects almost all organs. Cardiac involvement, in particular, may present a plethora of clinical manifestations such as pericarditis, myocarditis, valvular abnormalities, coronary heart disease, and conduction disorders. Vascular involvement such as vasculitis, thromboembolic disease, and microvascular angina has additionally been reported [1]. Up to 50% of SLE patients may have cardiac symptoms [2].

Conduction system disorders are less frequently reported in the currently available literature. Its actual prevalence remains largely undetermined. However, the clinical implications of these disorders are notable as arrhythmogenesis is attributed to the second mortality peak among SLE patients, potentially leading to sudden cardiac deaths [3,4]. A 20-year follow-up study by Abu-Shakra and colleagues showed that sudden death is the fourth leading cause of death in their SLE cohort [5]. A prevalence study of one center showed that sinus tachycardia (18%) was the most common ECG abnormality, followed by atrial fibrillation (9%) and prolonged QT interval (17%) [6]. For bradyarrhythmias, AV blocks, intraventricular conduction defects, and SND have been elucidated [4].

SND in SLE patients is rarely described. However, it could present as sinus bradycardia, sinus pause with junctional rhythm, sino-atrial block, chronotropic incompetence, and alternating bradycardia and tachycardia episodes (bradycardia-tachycardia syndrome) [7]. Case reports have been documented on SLE presenting with SND, but pertinent in all their profiles were the associated symptoms of fever, myalgia, arthralgia/arthritis, photosensitivity, malar rash, oral ulcers, and hair loss, leading to high suspicion for lupus [7,8]. SND with severe bradycardia in another report was noted to be associated with high disease activity [9]. In our patient, she presented with only vague bouts of fatigue, acute onset of left upper weakness, and an incidental finding of sinus bradycardia and atrial fibrillation. The patient met the 2012 SLICC criteria only on further laboratory workup.

The insufficient information on arrhythmias in SLE has made it difficult to determine its exact mechanisms. Some of the few proposed processes include the inflammatory milieu induced by pericarditis and myocarditis, atherosclerotic myocardial ischemia, or small-vessel vasculitis with associated collagen deposition and fibrosis, which subsequently impair the conduction system [4]. A vasculitis process selectively affecting the cardiac conduction tissue without associated myocarditis or cardiomyopathy has been proposed [8].

In our present case, high-dose steroid therapy was given for five days with the persistence of SND. Conduction tissue changes in SLE may be permanent despite high-dose steroids, necessitating pacemaker insertion as the definitive management [4]. Based on the 2021 European Society of Cardiology Guidelines on cardiac pacing and cardiac resynchronization therapy, our patient meets the indication for pacing in SND since our patient is symptomatic with the bradycardia-tachycardia form of SND [10].

Neurologic involvement in SLE can present in 24%-51% of cases. ATM/, however, is an uncommon but severe complication with an estimated prevalence of 1%-2% [11]. The pathogenesis of SLE-ATM has been thought of as a multifactorial consequence of autoantibodies, cytokines, and vasculitis processes leading to neuronal and axonal damage. A strong association between SLE-ATM and the presence of antiphospholipid antibodies (aPL) has been noted. Transverse myelitis in SLE can present as an acute motor, sensory or autonomic impairment, particularly progressive paraparesis, quadriparesis, paresthesia, and urinary and fecal incontinence [11,12].

In contrast, our patient presented with left arm weakness, paresthesia, and a single episode of urinary retention. In a case series by D'Cruz and colleagues, arm monoparesis/paresthesia was observed in only two out of 15 patients, with most cases initially presenting with spastic quadriparesis [13]. Transverse myelitis in SLE is well known as a late complication. However, a few investigations have documented it as the presenting manifestation and even taking place in the absence of active lupus activity, as is the case of our patient [12].

When SLE-ATM is a consideration, spinal cord MRI is the imaging of choice for diagnosis and follow-up. It is essential to rule out other possible etiologies of secondary myelitis such as infections (viruses, bacteria, parasites, fungi), malignancy, and other autoimmune disorders such as multiple sclerosis, neuromyelitis optica, and myelin oligodendrocyte glycoprotein antibody-associated disease (MOGAD) [12]. Our patient was negative for infections in our spinal fluid work-up and was negative for CSF oligoclonal bands. Other autoimmune work-up was not pursued due to financial constraints. SLE-ATM was heavily favored by combining the patient's positive serology (ANA, C3 hypocomplementemia, anti-dsDNA), characteristic imaging findings, and multiorgan involvement (cardiac and neurologic).

Given the low incidence of SLE-ATM, evidence-based treatment is limited. However, an accepted consensus among experts is to immediately initiate high-dose IV steroids and cyclophosphamide to prevent further spinal cord damage and disease progression [12,14]. More than half of patients respond to therapy, with some achieving fair functional outcomes, especially with the addition of an immunosuppressant [15]. Given the high relapse rate of approximately 50%, these patients should ensure good adherence to maintenance therapy, usually consisting of oral steroids, hydroxychloroquine, and immunosuppressants [12,16].

Pertinent in this case is that of the patient meeting the 2012 SLICC classification criteria, but a point short if the 2019 EULAR/ACR classification criteria for SLE is utilized since neuropathy was excluded in the latter. A look into the development of this classification criteria would reveal that this four-phase process initially considered five candidate criteria in the neuropsychiatric domain, namely, delirium, psychosis, seizure, mononeuropathy, and cranial neuropathy (phase 3a). However, the experts in the study unanimously dropped the latter two (phase 3b), owing to the less value these contribute to the weighted SLE classification [17]. This highlights that atypical presentation of SLE, such as in our patient, requires a high index of suspicion to conduct a lupus workup. This also emphasizes the need for a thorough documentation of rare presentations and even establishing SLE case registries to determine its prevalence. Addressing this information gap can hopefully be relevant in developing an inclusive diagnostic criteria for SLE.

## Conclusions

Much remains to be understood regarding SLE-related SND and ATM, from its prevalence to pathogenesis and the necessary diagnostic approach to management. Recognition and documentation of SLE's rare but life-threatening presentations, such as SLE-ATM and SND, are essential to facilitate timely therapeutic interventions.
